# Asymmetric Flow Dynamics in a Pneumatically Actuated Polydimethylsiloxane (PDMS) Micropump: Numerical Simulation and Experimental Validation

**DOI:** 10.3390/mi17091027

**Published:** 2026-08-28

**Authors:** Pei-Pei Hsu, Jr-Lung Lin

**Affiliations:** Department of Mechanical and Automation Engineering, I-Shou University, Kaohsiung 84001, Taiwan

**Keywords:** micropump, asymmetric flow dynamics, fluid–structure interaction, larger deformation, pumping efficiency

## Abstract

This study presents a comprehensive numerical and experimental investigation on a pneumatically actuated polydimethylsiloxane (PDMS) micropump integrated with passive check valves (PCVs). Advanced three-dimensional (3D) fluid–structure interaction (FSI) simulations were conducted to capture the nonlinear large deformation of the membrane and elucidate the Newtonian flow dynamics. The maximum deformation calculated by the simulations was compared with the theoretical Timoshenko formula, demonstrating excellent agreement across the entire pressure range (3.0–100.0 kPa). The 3D FSI simulations revealed a considerable asymmetry in the flow dynamics between the suction and compression phases. Notably, at applied pressures exceeding 20.0 kPa, the discharge volume substantially outweighed the suction one. To characterize both the ideal and practical volumetric flow rates, curve-fitting analyses revealed that both the simulation and experimental data follow a consistent 1/3-power-law relationship with respect to the applied pressure. Experimentally, the micropump achieved a maximum volumetric flow rate of 2.6 mL/min at 70.0 kPa and 12.0 Hz. Furthermore, the micropump demonstrated a peak pumping efficiency of 56.95% at 35.0 kPa and 10.0 Hz relative to the ideal numerical baseline. By bridging idealized numerical bounds with experimental realities, this validated framework offers a robust predictive tool for optimizing flow asymmetry and pumping efficiency in advanced microfluidic systems.

## 1. Introduction

Micropumps have been integral to a wide spectrum of biomedical applications over the past decades [1]. These applications can be broadly categorized according to the necessary flow rates. In low-throughput scenarios, such as lab-on-a-chip systems and targeted drug delivery (e.g., for administering insulin), micropumps achieve highly precise fluid control [2,3]. Conversely, high-throughput micropumps are crucial for applications demanding rapid and larger volumetric fluid transport, such as ventricular assist devices [4,5]. Despite the progress in meeting these diverse requirements, a critical challenge remains in developing devices that ensure predictable dynamic performance while achieving the desired throughput, particularly when integrating advanced sensing, monitoring, and intelligent analytical frameworks for enhanced system reliability [6].

Mechanical micropumps comprise three fundamental components: an actuated diaphragm, a fluidic pump chamber, and a flow rectification mechanism. Typically, the diaphragm is driven by piezoelectric [7,8], electrostatic [9,10], electromagnetic [11,12], or pneumatic [13,14,15] actuation to perform reciprocating fluid suction and discharge. Flow rectification mechanisms are essential for breaking the spatial symmetry of these oscillations and generating unidirectional net flow. Based on these mechanisms, micropumps are broadly classified into valveless and valved architectures. Conventional valveless pumps, which utilize nozzle/diffuser elements [16,17], or Tesla channels [18], effectively minimize mechanical wear. However, across different scales of pump systems, from macro- to micro-scale fluidic devices, net efficiency is frequently compromised by complex internal flow phenomena. These include inherent dynamic backflow, internal liquid leakage, and high flow resistance [19,20]. In contrast, valved pumps incorporate either active or passive microvalves. While active valves enable precise flow manipulation, they require bulky external power sources and complex control circuitry [14,21,22]. To overcome these limitations and optimize integration while maintaining reliable pumping performance, passive check valves (PCVs), including normally closed [23], flap [24], ball [25], and umbrella designs [26], are preferable for flow rectification.

Previously, we successfully developed an active microvalve actuated by a pneumatically driven membrane [14] and a valveless micropump where a single diaphragm served as the actuator and valves [15]. These validation studies established large-deformation theories and 3D structural simulations for rectangular [14] and circular membranes [15,27], respectively. However, the analytical scope was strictly confined to pure solid mechanics, without accounting for fluid–structure interaction [28]. Simulating the FSI is essential for bridging this gap and capturing the actual physical phenomena. While FSI simulations are generally categorized into one- and two-way coupling approaches, one-way coupling—where only fluid pressure is transferred to the structural solver—inherently lacks dynamic feedback, despite reducing computational time and divergence issues [29]. Conversely, in a two-way coupling simulation, the structural deformation is continuously transferred back to the fluid solver to dynamically update the fluid domain. This bidirectional interaction is crucial for accurately modeling the complex dynamics of actuated membranes and valves for micropumps. Building upon our foundational models, the present study employs a fully coupled two-way formulation to achieve comprehensive and high-fidelity results for the transient behavior of a new pneumatic micropump. Additionally, geometric nonlinearity was explicitly considered in the structural numerical simulation to account for the large membrane deformation.

Despite extensive experimental developments, rigorous theoretical modeling and numerical simulations of transient dynamics in micropumps remain poorly understood. Previous modeling has relied on simplified lumped-parameter methods [11,30] or two-dimensional (2D) simulations [31]. Furthermore, even after employing 3D FSI simulations, they are typically restricted to small-scale membrane deflections (e.g., piezoelectric actuation) [32,33,34]. Thus, conventional approaches fail to accurately represent the complex FSI inherent in highly nonlinear, large-deformation pumping. This extreme geometric nonlinearity extensively hinders deriving exact analytical correlations for instantaneous flow rates. Similarly, resolving these coupled mechanics through 3D numerical simulations is significantly hindered by considerable computational complexity and severe convergence instabilities [28,29,32]. Although some recent studies have explored 3D FSI for valved micropumps [33], the literature integrating these advanced simulations with rigorously derived nonlinear integral models and empirical validations remains underdeveloped.

To bridge these critical gaps, this study introduces a streamlined, highly manufacturable four-layer polydimethylsiloxane (PDMS) micropump, equipped with PCVs, as a reliable physical platform. Based on this design, we demonstrate and validate comprehensive 3D FSI numerical simulations. This approach systematically elucidates the fully coupled mechanisms between structural membrane deformation and fluidic pumping dynamics. The primary contribution of this work lies in rigorously quantifying the dynamic correlation between nonlinear PDMS membrane kinematics and transient flow behaviors. By empirically validating the numerical method, this study provides a powerful and highly efficient predictive tool for the precise design and optimization of future advanced micropumps.

## 2. Numerical Modeling

### 2.1. Computational Framework and Material Properties

To systematically optimize the design and evaluate the transient dynamics of the pneumatically actuated PDMS micropump, a comprehensive 3D FSI numerical model was developed using a commercial code (CFD-ACE+, CFDRC, Huntsvile, AL, USA) [14,15]. The system was governed by a fully coupled two-way FSI framework that simultaneously solved the unsteady continuity and Navier–Stokes equations for the fluid domain and the nonlinear elastodynamic equations using the Saint-Venant Kirchhoff (SVK) model for the solid structure. To accurately represent the physical system, the assigned material properties of the PDMS membrane included a density of 970 kg/m^3^, Young’s modulus of 2.0 MPa, and Poisson’s ratio of 0.499 [15]. Additionally, the working fluid (water) was defined as an incompressible Newtonian fluid with a density of 1000 kg/m^3^ and dynamic viscosity of 1.0 mPa·s. To avoid prohibitive computational costs, this 3D FSI framework specifically focused on the primary dynamic coupling between the actuated membrane and the pump chamber fluid.

The entire 3D computational domain was discretized using structured hexahedral meshes, with localized grid refinement applied near the highly deformed regions. To accommodate the severe membrane displacements and maintain computational stability, an auto-remeshing algorithm was employed to dynamically update the moving boundaries. To ensure the numerical reliability of the simulation under these dynamic conditions, grid and temporal convergence studies were conducted. A grid convergence study across three mesh densities (279,536, 397,776, and 516,016 cells) demonstrated that the velocity profiles and volumetric flow rate achieved satisfactory asymptotic convergence at approximately 400,000 cells. Additionally, temporal convergence verified that a standard time step of 6.25 ms (millisecond) was optimal for general operations, while a refined time step of 3.125 ms was dynamically implemented for higher pressures (≥20 kPa) to accurately resolve transient membrane dynamics during contact and bottoming phases (see Supplementary Document S1 for details).

### 2.2. Governing Equations

The following sections present the specific formulations governing the fluid domain, solid structure, and fluid–solid interface in detail [35].

For the incompressible Newtonian fluid domain, the continuity and momentum (Navier–Stokes) equations are given by:

(1) $$\frac{\partial u_{f}}{\partial t}+\rho_{f}\nabla \cdot u_{f}=0$$

and

(2) $$\rho_{f}\frac{\partial u_{f}}{\partial t}+\left(u_{f}\cdot \nabla \right)u_{f}=\nabla \cdot \left[{-P}_{f}I+\mu \left(\nabla u_{f}+{\left(\nabla u_{f}\right)}^{T}\right)\right]+F_{ext}$$

where *u_f_* is the fluid velocity vector, *ρ_f_* is the fluid density, *P_f_* is the fluidic pressure, *μ_f_* is the dynamic viscosity, and ***F****_ext_* represents the external force vector per unit volume. The symbol *I* denotes the identity tensor, and the superscript *T* denotes the transpose.

The solid mechanics module defines the PDMS membrane as an isotropic and linearly elastic material undergoing large deformations. The local equilibrium, essential for capturing transient dynamic responses, is governed by

(3) $$\rho_{s}\frac{\partial^{2}u_{s}}{\partial t^{2}}=\nabla \cdot {\left(F_{ext}S\right)}^{T}+B$$

where *u_S_*, *ρ_s_*, **B**, and **S** represent the solid velocity vector, solid density, body force vector per unit volume, and second Piola–Kirchhoff stress tensor, respectively.

To accurately capture the geometric nonlinearity induced by large membrane deflections, the deformation gradient tensor, *F_V_*, is introduced to map the material configuration:

(4) $$F_{V}=I+\nabla u_{s}$$

where ∇*u_s_* denotes the displacement gradient. Consequently, the Green-Lagrangian strain tensor, ε, is introduced to fundamentally filters out rigid-body rotations to measure pure structural deformation:

(5) $$\varepsilon =\frac{1}{2}\left[{\left(\nabla u_{S}\right)}^{T}+\nabla u_{S}+{\left(\nabla_{S}\right)}^{T}\right]$$

The constitutive response of the soft material assumes an isotropic linear elastic body described by the St. Venant–Kirchhoff (SVK) model, which ensures robust 3D FSI convergence for the bending-dominated large displacements in this study. Accordingly, the second Piola–Kirchhoff stress tensor, S, is explicitly formulated in terms of the Green-Lagrangian strain tensor, ε, through the generalized Hooke’s law:

(6) S=λ⋅tr(ε)I+2G⋅ε

where λ represents the first Lamé parameter, G the shear modulus of the solid material, and tr() the trace operator.

At the fluid–solid interfaces, kinematic and dynamic continuity conditions are strictly enforced to capture the continuous bidirectional feedback between the instantaneous fluidic pressure and the large-scale structural deformation. The fluid–solid interaction boundary equations are expressed as

(7) $$F_{A}=\left[-\rho_{f}I+\mu \left(\nabla u_{f}+{\left(\nabla u_{f}\right)}^{T}\nabla u\right)\right]\cdot n$$

(8) $$u_{tr}=\frac{\partial u_{S}}{\partial t}$$

where *F_A_* represents the total hydrodynamic force exerted by the fluid on the fluid–solid interface, *n* is the outward unit normal vector, and *u_tr_* denotes the rate of change in the solid displacement (i.e., the interface velocity).

### 2.3. Initial and Boundary Conditions

To simulate the active pneumatic actuation explicitly, a time-dependent sinusoidal pressure function, *P_a_*, was applied to the actuated membrane surface:

(9) $$P_{a}=P_{o}\sin(2\pi ft)$$

where *P_o_* represents the applied pressure amplitude and *f* the driving frequency.

The simulation was initiated from a state of rest, without structural displacement and fluid velocity. Standard impermeable, no-slip boundary conditions were applied to all rigid fluidic walls. Regarding the structural domain, the periphery of the circular membrane was rigidly clamped (zero displacement). A pneumatic pressure was imposed on the membrane’s top surface, while its bottom surface was allowed to freely deform to interact with the fluid. The flow was driven by this pneumatically actuated membrane against a zero-gauge-pressure opening. As a limitation of the current 3D FSI framework, the PCVs were modeled using idealized, synchronized wall-switching boundary conditions to simulate directional flow and strictly prohibit backflow. Specifically, during the suction half-cycle, the outlet boundary was instantaneously defined as a stationary “wall” (zero velocity) while the inlet remained open(zero gauge pressure); conversely, during the discharge half-cycle, the inlet was defined as a “wall” while the outlet was open. This alternating approach intentionally neglects the physical limitations of real-world check valves—such as opening/closing delays, structural inertia, valve deformation, and dynamic backflow—thereby establishing an ideal (theoretical) ‘upper-limit’ flow rate as a baseline for comparison against experimental performance.

## 3. Materials and Method

### 3.1. Design and Working Principle

**Figure 1:**
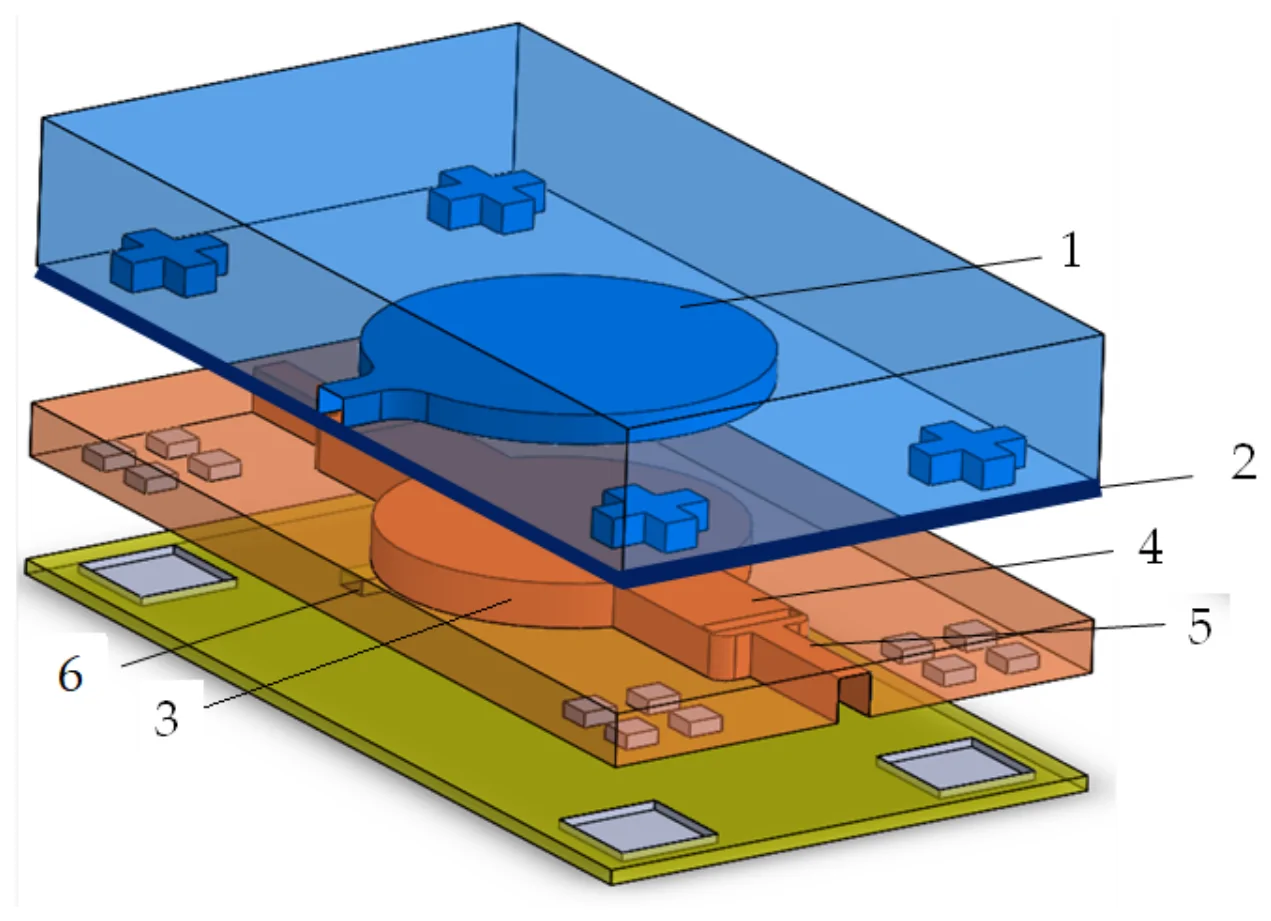
Exploded view of the proposed micropump: (1) air chamber; (2) actuated membrane; (3) fluidic pump chamber; (4) fluidic channel 1; (5) fluidic channel 2; (6) passive check valve.

The proposed micropump was constructed by laminating four distinct PDMS layers (Figure 1). These layers comprise the pneumatic air chamber, the actuated flexible membrane, the fluidic pump chamber (featuring specific channel designs), and the bottom substrate housing two PCVs. The air and fluidic chambers are 1.0 and 1.5 mm thick, respectively, with both sharing a diameter of 10.0 mm. Between them is a 300-μm-thick flexible membrane measuring 30.0 × 20.0 mm^2^, which is driven by pneumatic pressure. Notably, the integrated PCVs are housed within Channel 1 (3.0 mm wide), with one positioned at the exit of the pump chamber and the other located at the junction with the narrower Channel 2 (1.5 mm wide). The difference in cross-sectional areas serves as a physical stopper to inherently prevent backflow during the compression or suction phase, achieving a monolithic sealing design without requiring conventional solid stoppers. The geometrical specifications of the micropump are listed in Table 1.

**Figure 2:**
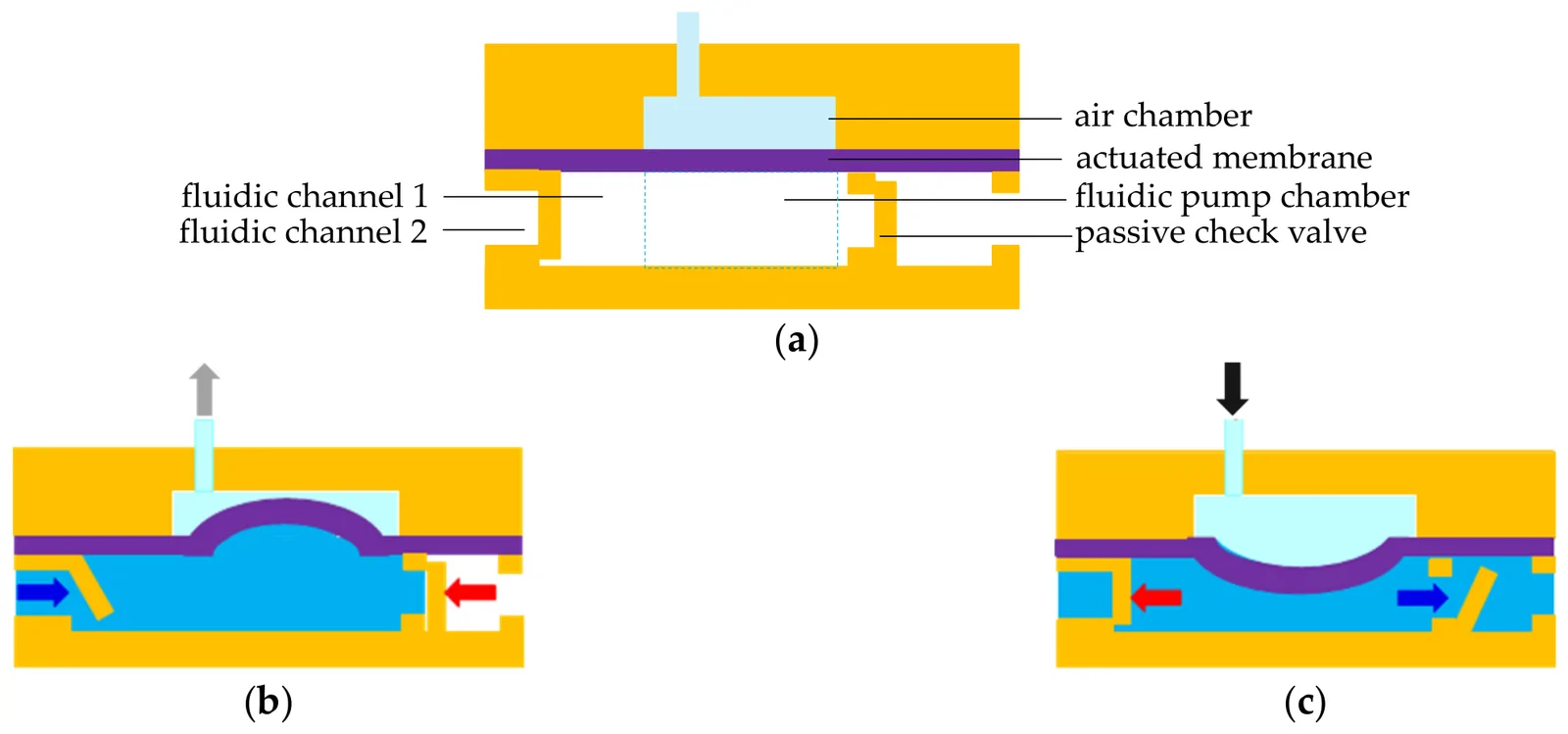
(**a**) Schematic front view of the proposed micropump, illustrating the working principles during the (**b**) suction process (gray arrow) and (**c**) compression process (black arrow). The inlet and outlet PCVs regulate the fluid direction by opening to permit forward flow (blue arrows) and closing to prevent backflow (red arrows).

Figure 2 schematizes the structure of the proposed micropump (Figure 2a) and demonstrates the working principle of the actuated membrane through two sequential processes: suction and compression. The design incorporates two PCVs with specifically designed cross-sectional geometries that act as physical stoppers to regulate the flow direction. Initially, the fluidic pump chamber is unpressurized when the actuated membrane is in its resting, i.e., unactuated state. During the suction process (Figure 2b), negative pneumatic pressure pulls the actuated membrane upward. This upward deflection creates a low-pressure environment within the fluidic pump chamber, which opens the inlet check valve (left) while simultaneously closing the outlet check valve (right) shut against its stopper. Consequently, liquid is drawn in through the inlet port to fill the fluidic pump chamber.

During the compression process (Figure 2c), positive pneumatic pressure pushes the actuated membrane downward. This downward deflection compresses the liquid within the fluidic pump chamber, forcing the inlet check valve (left) closed against its stopper to prevent backflow. Simultaneously, the pressurized fluid pushes the outlet check valve (right) open, allowing the liquid to be directly expelled through the outlet port. By continuously alternating the upward pull (suction) and downward push (compression) of the actuated membrane at a given driving frequency, the liquid is steadily transported from the inlet to the outlet, thereby achieving an effective and continuous pumping operation.

### 3.2. Chip Fabrication

**Figure 3:**
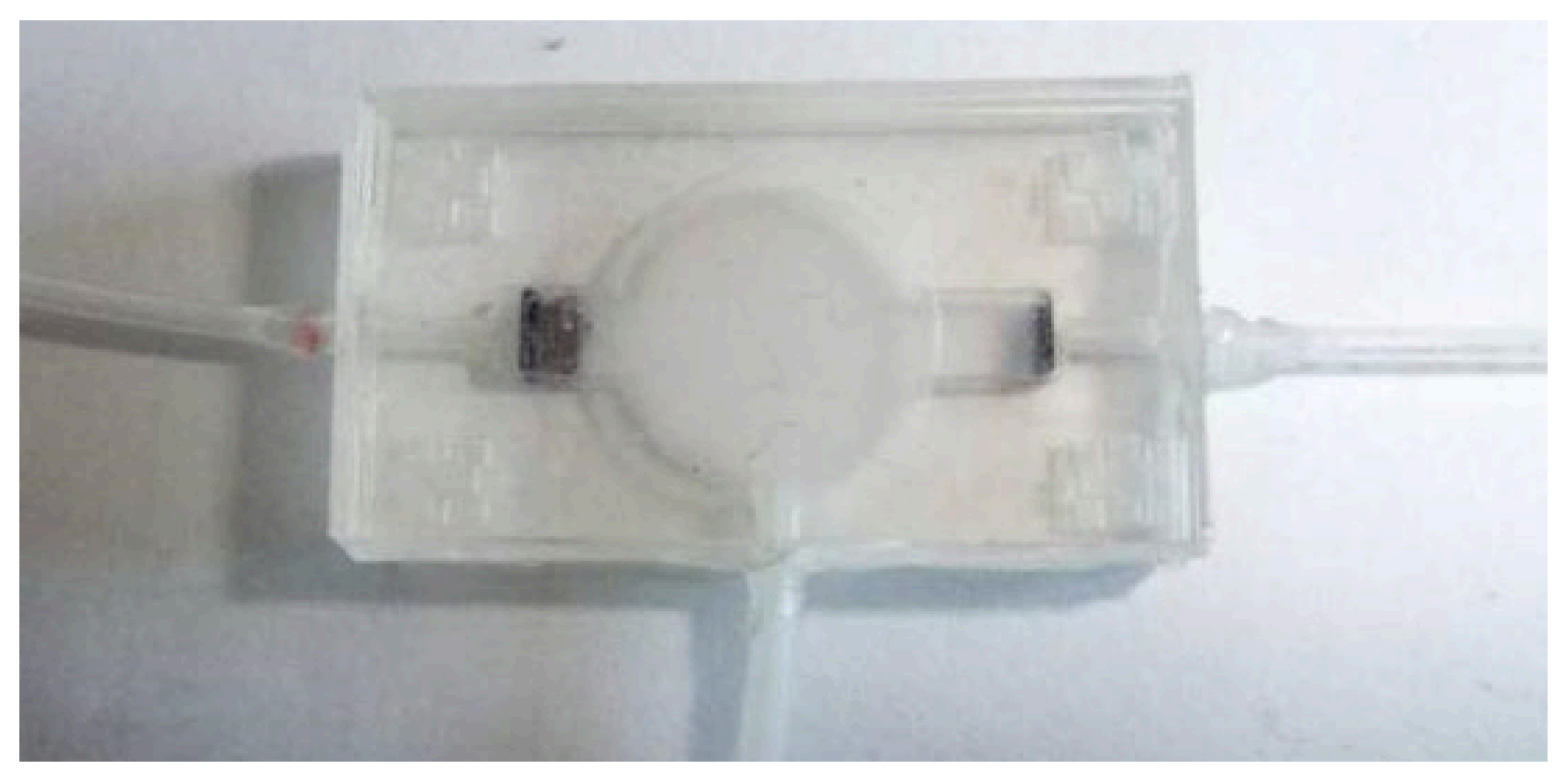
Prototype photograph of the assembled PDMS micropump, with the integrated PCVs appearing in black.

The proposed micropump was fabricated using standard soft-lithography techniques. Initially, master molds featuring the inverse microstructures were precisely milled into polymethyl methacrylate (PMMA) (Rixiang Acrylic, Kaohsiung City, Taiwan) substrates using a computer numerical control (CNC) machine (EGX-400, Roland Inc., Hamamatsu, Japan). A liquid PDMS (Sylgard 184, Dow Corning, Midland, MI, USA) prepolymer mixture was then poured over the molds, degassed, and cured to replicate the designated geometries. To form the monolithic pump assembly, the four PDMS layers were sequentially aligned and irreversibly sealed together through oxygen plasma activation. A prototype photograph of the assembled PDMS micropump is displayed in Figure 3.

### 3.3. Experimental Setup

The experimental platform for assessing the capabilities of the micropump was equipped with a custom control circuit, two electromagnetic valves (EMVs) (s070 m-5bg-32, SMC Inc., Taoyuan City, Taiwan), and a pneumatic air compressor (mdr2-1a/11, JUN-AIR Inc., Tokyo, Japan) [15]. The device was systematically investigated by monitoring the flow output across a range of driving frequencies and applied pneumatic actuation pressures. Throughout the testing phase, the dynamic fluid transport was continuously captured visually using a digital camera (E-5P, Olympus, Tokyo, Japan). To precisely quantify the pumping performance, the liquid discharged over a 1-min period was weighed on an analytical balance (AB54-S, Mettler Toledo, Taipei City, Taiwan). These mass measurements were subsequently translated into volumetric flow rates by applying a standard deionized (DI) water density of 1.0 g/cm^3^ at room temperature of 25 °C.

## 4. Results and Discussion

### 4.1. Numerical and Theoretical Validation of Structural Behavior

**Figure 4:**
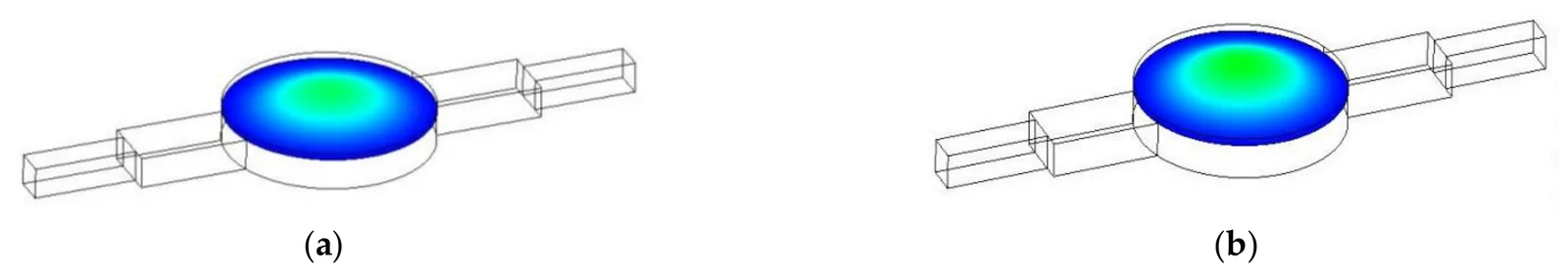

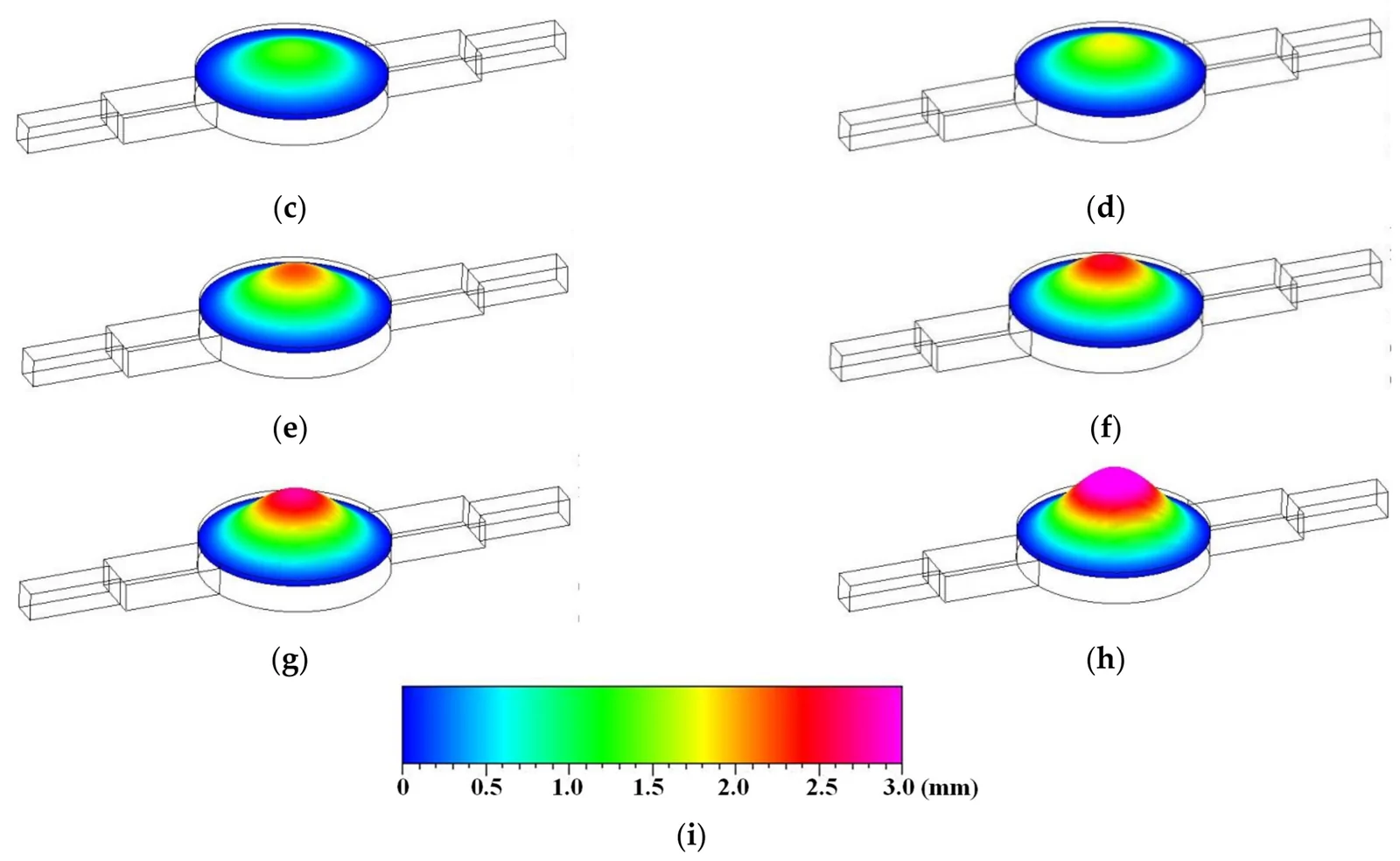
Numerical deformation profiles of the actuated membrane under different applied pneumatic pressures: (**a**) 3.0, (**b**) 5.0, (**c**) 10.0, (**d**) 20.0, (**e**) 40.0, (**f**) 60.0, (**g**) 80.0, and (**h**) 100.0 kPa. (**i**) deformation contour color bar.

Figure 4 presents the 3D numerical simulation results, illustrating the cross-sectional deformation profiles of the PDMS membrane under varying applied pneumatic pressures. The colored surface plots clearly exhibit a symmetrical, dome-shaped deflection pattern, where the maximum vertical displacement occurs precisely at the center while the perimeter remains firmly clamped. The numerical data reveal that, as the driving pressure increases sequentially as 3.0, 5.0, 10.0, 20.0, 40.0, 60.0, 80.0, and 100.0 kPa, the corresponding maximum deflections are recorded as 0.96, 1.15, 1.47, 1.80, 2.27, 2.57, 2.81, and 3.07 mm, respectively. Notably, analyzing the deflection increments reveals a distinct geometric nonlinearity in the membrane’s structural behavior. In lower pressure regimes (3–40.0 kPa), the membrane deforms rapidly in response to pressure changes, maintaining high deflection increment rates ranging between 19.8% and 27.8%. However, as the driving pressure exceeds 40.0 kPa, the increment rate begins to decline progressively, dropping to 13.2% at 60.0 kPa. Ultimately, the increment rate approaches a saturation plateau of exactly 9.3% at both 80.0 kPa and 100.0 kPa. This progressive stiffening effect indicates that the stretching strain energy begins to dominate the bending energy at large deformations. These comprehensive simulation results verify the stable and predictable actuation behavior of the PDMS membrane, serving as a critical factor for determining the dynamic stroke volume of the proposed micropump.

The large membrane deflection was predicted using a classic Timoshenko formula [36], yielding the following relationship:

(10) $$\frac{w_{o}}{a}=0.662{\left(\frac{P_{o}a}{Eh}\right)}^{\frac{1}{3}}$$

This formulation predicts the maximum membrane deformation based on its geometry and material properties. By substituting the specific parameters of the designed micropump (Young’s Module *E* = 2.0 MPa, membrane radius *a* = 5 mm, and thickness *h* = 300 μm) into Equation (11), the theoretical relation under *P_o_* simplifies to a concise analytical form for direct numerical comparison:

(11) $$w_{o}=0.671\cdot P_{o}^{1/3}$$

where *P_o_* is the applied pressure (kPa), and *w_o_* represents the maximum center deflection (mm). This simplified explicit theoretical equation provides a clear mathematical baseline to evaluate the accuracy of the established 3D FSI simulation model.

**Figure 5:**
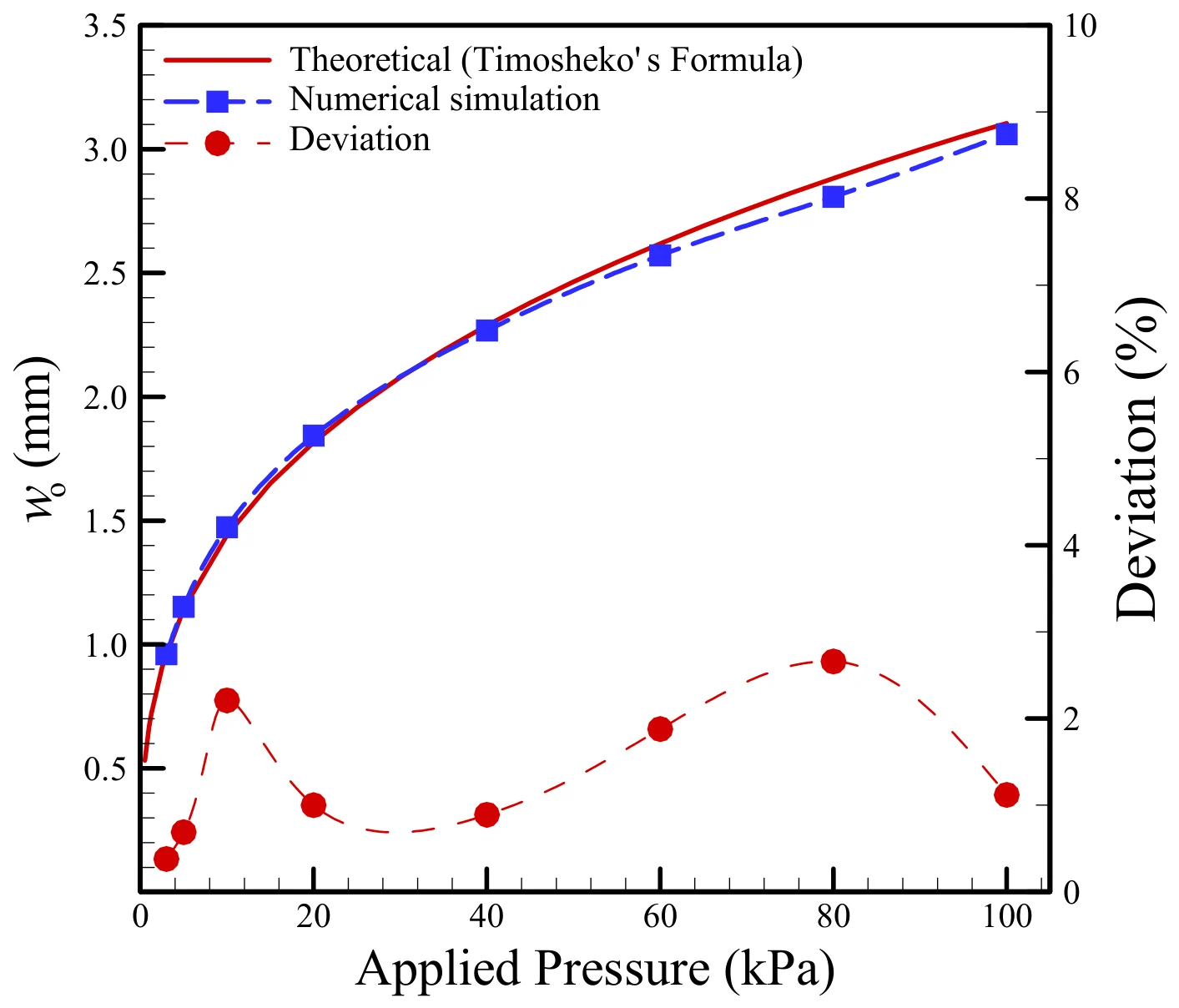
Quantitative comparison of the maximum membrane deformation (*w_o_*) obtained from 3D numerical simulations and the theoretical Timoshenko formula (Equation (11)) under varying applied pressures.

To ensure reliability, a two-step validation strategy was employed: first validating the structural baseline via the Timoshenko formula to ensure accurate membrane kinematics, and subsequently validating the overall fully coupled 3D FSI framework by comparing the predicted volumetric flow rates with experimental measurements. Figure 5 compares the numerical results with Equation (11), demonstrating excellent agreement across the entire pressure range (3.0–100.0 kPa). Notably, the relative deviation between the simulated results and theoretical predictions remains strictly below 3% across all examined pressure levels. While the theoretical model provides a convenient and efficient estimation of membrane deflection, it inherently cannot capture the complex, transient 3D FSI dynamics within the micropump. Understanding the intricate coupling mechanisms between the large membrane deformation and the internal flow field necessitates advanced 3D FSI numerical simulations. Therefore, the good agreement with the classic Timoshenko formulation serves as a crucial first validation step. It rigorously verifies the structural accuracy of the 3D model, effectively justifying the computational approach and establishing the reliability of the framework for the subsequent 3D FSI flow analyses.

### 4.2. Fluidic-Structural Dynamics and Flow Asymmetry

**Figure 6:**
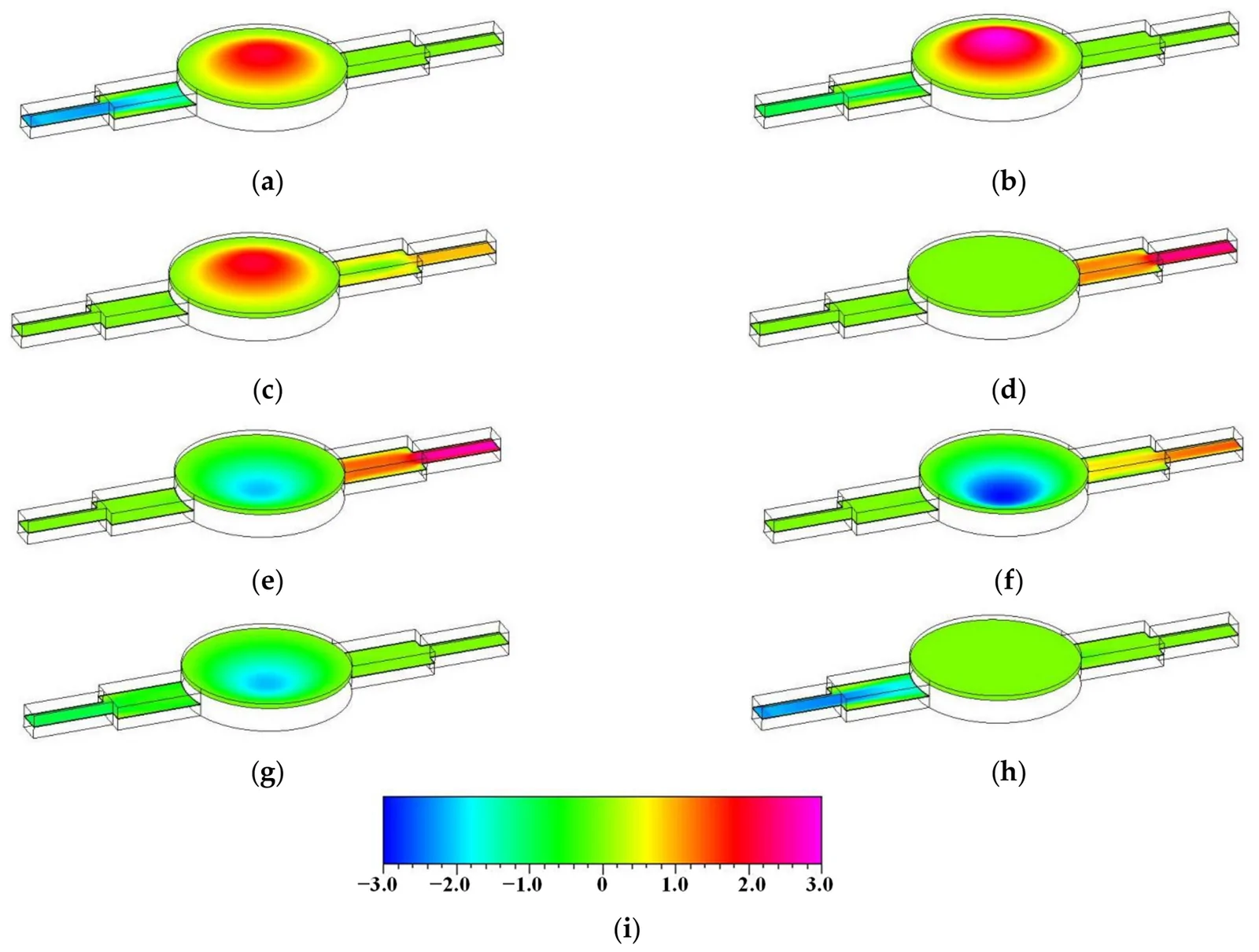
Transient numerical simulation of the actuated membrane deformation and the corresponding fluid velocity field at 10.0 kPa and 10.0 Hz. The sequential snapshots represent different time steps within a complete pumping cycle (T = 100 ms): (**a**) t = 1.25 ms; (**b**) t = 25 ms; (**c**) t = 37.5 ms; (**d**) t = 50 ms; (**e**) t = 62.5 ms; (**f**) t = 75 ms; (**g**) t = 87.5 ms; and (**h**) t = 100 ms. (**i**) Velocity magnitude color bar.

**Figure 7:**
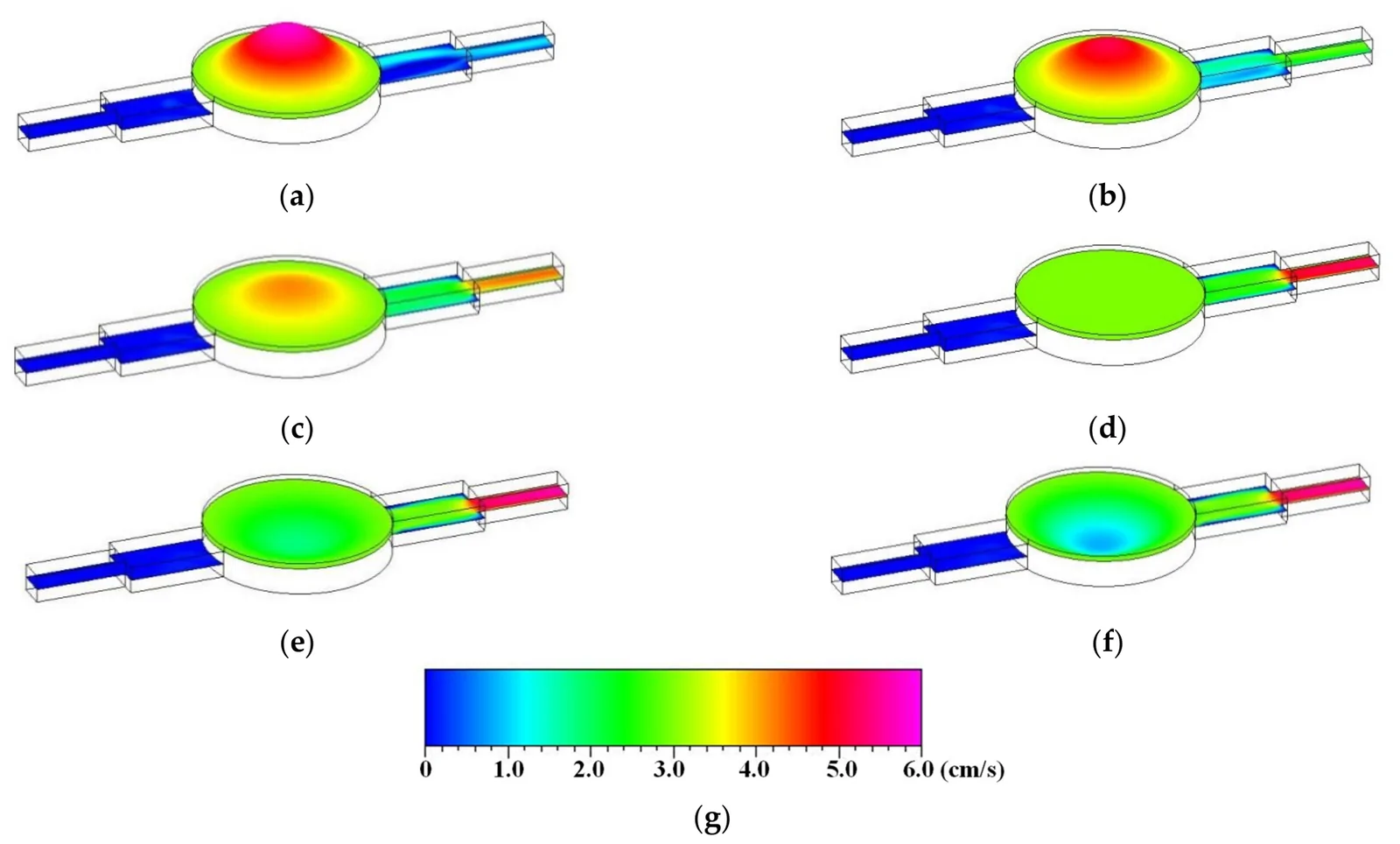
Transient numerical simulation of the actuated membrane deformation and the corresponding fluid velocity field at 100.0 kPa and 10.0 Hz. The sequential snapshots represent different time steps within a compression half-cycle: (**a**) t = 25.0 ms; (**b**) t = 31.25 ms; (**c**) t = 43.75 ms; (**d**) t = 50.0 ms; (**e**) t = 54.69 ms; and (**f**) t = 59.38 ms. (**g**) Velocity magnitude color bar.

To evaluate the dynamic transportation performance systematically, 3D FSI numerical simulations were conducted under varying applied pressures at a constant driving frequency of 10.0 Hz. The depth of the fluidic pump chamber was strictly constrained to 1.5 mm to match the exact geometric dimensions of the real-world prototype, thereby accurately capturing its spatial confinement and the associated fluidic resistance in the numerical model. To elucidate the internal pumping dynamics, the sequential evolution of the transient membrane deformations and their corresponding fluidic flow fields are illustrated in Figure 6 (for a complete operating cycle) and Figure 7 (for a compression half-cycle). In the velocity fields presented for a complete cycle, the color scheme notably denotes the operational phases: blue represents the suction velocity, while red represents the compression (discharge) velocity. This color mapping specifically distinguishes the fluidic directional response induced by the membrane’s motion, rather than merely indicating positive or negative scalar values.

Figure 6 presents the FDS pumping dynamics under an applied pressure of 10.0 kPa and a driving frequency of 10.0 Hz. At this pressure, the membrane deforms maximally by 1.47 mm. Because this value is less than the physical chamber depth of 1.5 mm, the membrane smoothly completes a full, unconstrained pumping cycle. A distinct kinematic-fluidic correlation is observed: the maximum velocities occur precisely when the membrane passes through its neutral equilibrium position, reaching approximately 2.0 cm/s during the suction phase (Figure 6a,h), and 3.0 cm/s during the discharge phase (Figure 6d). Conversely, the fluid velocities drop to approximately zero at the points of maximum membrane deflection, i.e., at the top dead center of the suction phase (Figure 6b) and the bottom dead center of the compression phase (Figure 6f). Since the membrane dynamics during the suction phase at 100.0 kPa closely resemble those observed in the 10.0 kPa case, the suction process is not further detailed here. Instead, this discussion focuses exclusively on the downward stroke (compression phase), as the extreme deformation during this phase induces a physical truncation that directly limits the discharge volumetric flow rate. Figure 7 illustrates the dynamics during the downward stroke (from the highest position to the lowest point) under a much higher applied pressure of 100.0 kPa and a driving frequency of 10.0 Hz. Under this condition, the theoretical unconstrained deformation of the membrane would in principle reach 3.07 mm, significantly exceeding the 1.5 mm geometric limitation of the chamber. Consequently, the 3D FSI simulation accurately captures a physical truncation: the membrane forcefully compresses against the chamber floor at t = 59.38 ms (Figure 7f). At this critical contact point, the numerical calculation must be strictly terminated. Continuing the simulation beyond this physical limit would induce severe mesh distortion, generating negative cell volumes and inevitably causing the FSI solver to diverge. While the velocity-position correlation remains consistent—with peak velocities at the equilibrium position (Figure 7d) and minimal velocities at the top and bottom dead centers (Figure 7a,f)—this premature bottom-out phenomenon effectively curtails the discharge stroke, fundamentally altering the high-pressure fluidic delivery.

**Figure 8:**
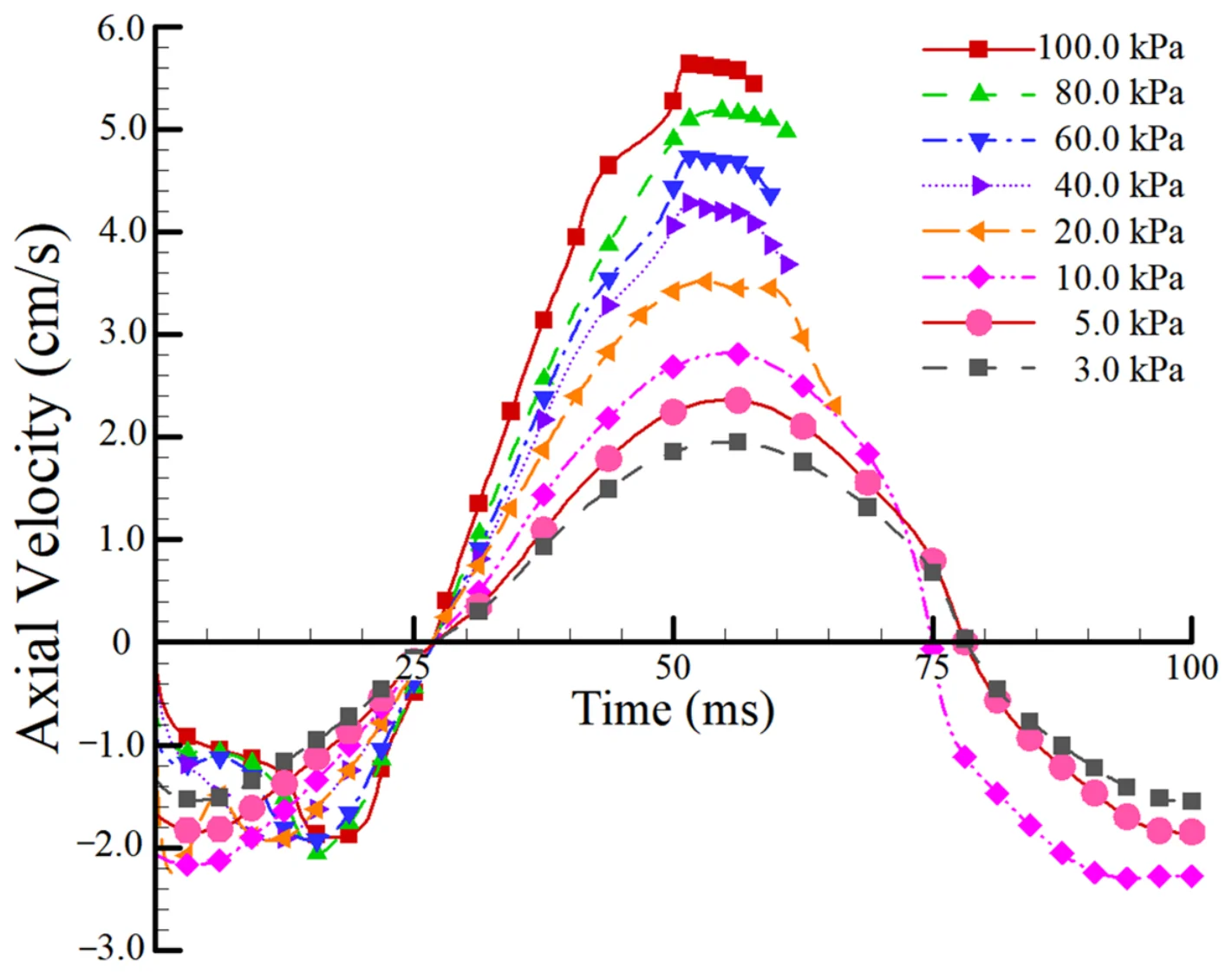
Transient outlet velocity profiles under varying applied pressures, demonstrating the symmetric flow dynamics in the low-pressure regime (≤10.0 kPa) and asymmetric flow dynamics in the higher-pressure regime (≥20.0 kPa).

Figure 8 plots the transient axial velocity profiles at the channel outlet, highlighting a fundamental shift in fluidic behavior between the low- and high-pressure regimes. For an incompressible fluid, the instantaneous volumetric flow rate (Q) is fundamentally governed by the rate of chamber volume change (dV/dt). This relationship can be expressed as Q = dV/dt = ŪA, where Ū is the cross-sectional average velocity and A is the channel area. Consequently, integrating the velocity-time curve over a phase (e.g., suction or compression) and multiplying it by the cross-sectional area yields the total stroke volume, while its scaled time-averaged value corresponds to the volumetric flow rate.

In the low-pressure regime (≤10.0 kPa), the maximum downward membrane deflection remains less than the 1.5-mm chamber depth, allowing a full, unconstrained kinematic cycle. Under this condition, the maximum suction and discharge velocities proportionally increase with the applied pressure, yielding balanced volumes, as confirmed by their identical time integrals. Conversely, a distinct dynamic asymmetry emerges in the high-pressure regime (≥20.0 kPa) due to imbalanced driving forces. During the discharge phase, the active pneumatic pressure drives a rapid dV/dt, producing a sharp flow-rate peak that increases significantly with pressure. In contrast, the suction phase relies solely on the membrane’s passive elastic restoring force. This restricted force limits the achievable dV/dt against inlet resistance, driving the system into a ‘suction-limited’ regime where the maximum suction velocity saturates at approximately 2.0 cm/s regardless of applied pressure. Consequently, the drawn fluid volume becomes substantially smaller than the expelled volume, yielding a distinctly asymmetric net flow.

Furthermore, these powerful active discharges accelerate the membrane to forcefully contact the chamber substrate progressively earlier in the compression phase. To prevent numerical divergence, simulations are intentionally terminated at these contact times (observed as the abruptly truncated curves after t = 50 ms in Figure 8). Once this contact threshold is reached, the effective stroke volume is geometrically truncated, preventing further flow-rate gains. Overall pumping performance is further degraded by rigid impact energy loss, severe squeeze-film damping, and the aforementioned suction limitations delaying the recovery stroke. Since the extruded residual volume from real-world post-contact effects is negligible compared to the primary stroke, these high-pressure flow characteristics reflect genuine physical phenomena rather than termination artifacts. Incorporating full-contact 3D FSI to capture these micro-scale post-contact mechanics remains future work.

### 4.3. Comprehensive Validation of Pumping Performance

**Figure 9:**
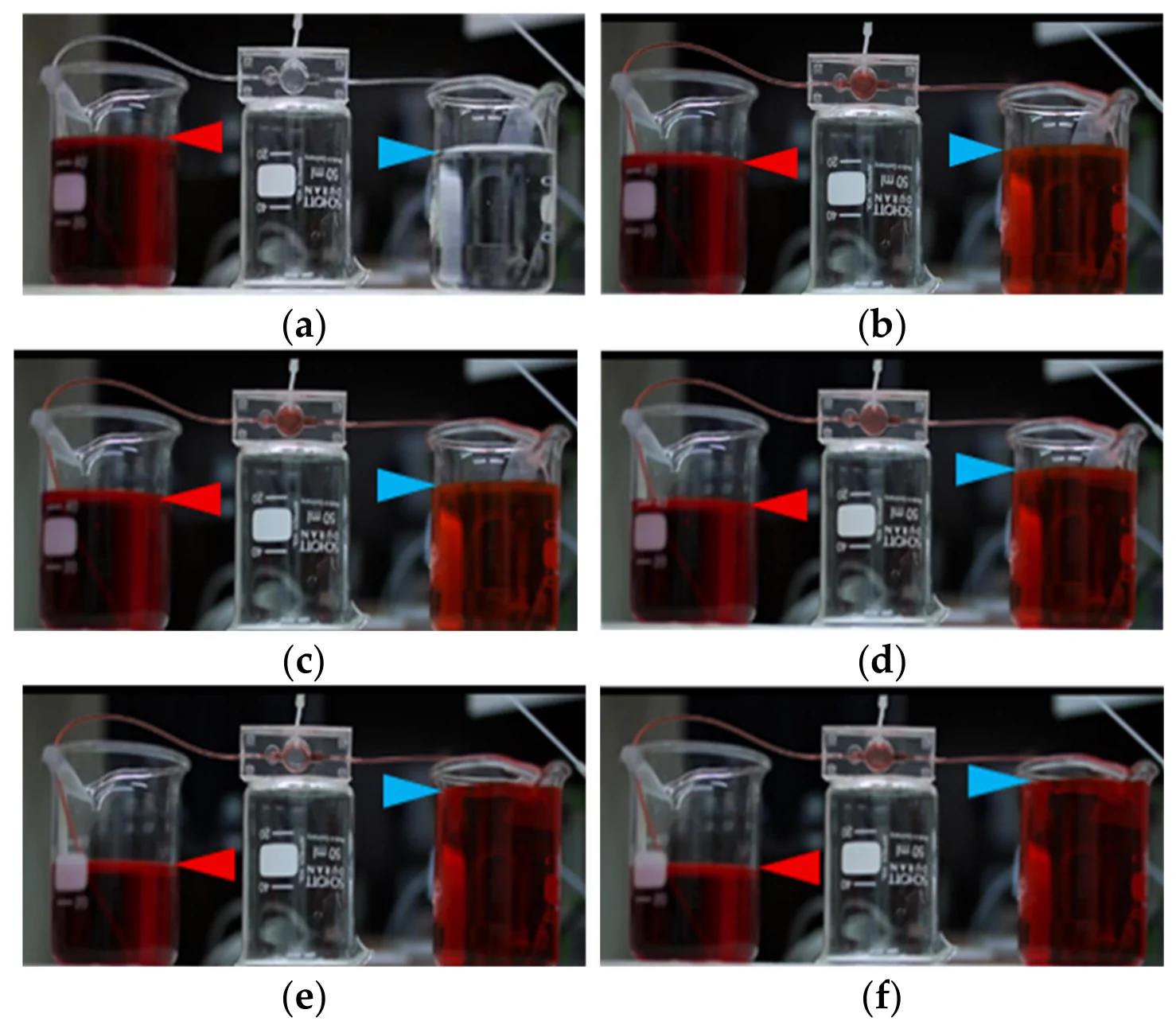
Sequential photographs illustrating the macroscopic continuous pumping process of the proposed micropump at (**a**) t = 0, (**b**) t = 2.0, (**c**) t = 4.0, (**d**) t = 6.0, (**e**) t = 8.0, and (**f**) t = 10.0 min. Red and blue tracking arrows indicate the dynamic liquid level changes.

To visually demonstrate the transportation capability of the proposed micropump, a macroscopic pumping experiment was conducted, as shown in Figure 9. The experimental setup consisted of a source beaker filled with red-dyed water (left) and collection beaker initially containing clear water (right). To eliminate any gravity-driven siphon effect, the pump was elevated on a central stand above the liquid levels of both beakers. Before conducting the measurement, the system was primed with liquid to eliminate internal air bubbles and ensure accuracy. To verify the long-term operational stability of the proposed micropump, the pumping experiment was continuously conducted over 10 min. The mass of the transported liquid was recorded at 1-min intervals. The mass of the transported water converted into volumetric flow rate. Figure 9a illustrates the initial state prior to actuation. Once the membrane was pneumatically driven at 70.0 kPa and 10.0 Hz, the red liquid was continuously drawn from the left beaker and discharged into the right beaker. To visually demonstrate this continuous pumping capability, the sequential photographs (Figure 9b–f) capture the macroscopic process at 2-min intervals over a total duration of 10 min. The red and blue tracking arrows, referenced against the beaker’s standard 50 mL scale, highlight a cumulative liquid displacement of approximately 26.0 mL (Figure 9f). This dynamic process explicitly confirms the effective suction and compression cycles of the actuated membrane, yielding a steady-state volumetric flow rate of 2.6 mL/min (see Supplementary Video S2). At this maximum flow rate (2.6 mL/min, 12.0 Hz), a Reynolds number (Re) of ~29 confirms strictly laminar, viscous-dominated flow. Meanwhile, a Strouhal number (St) of ~0.93 indicates significant unsteady oscillatory effects, justifying our transient dynamic modeling approach.

**Figure 10:**
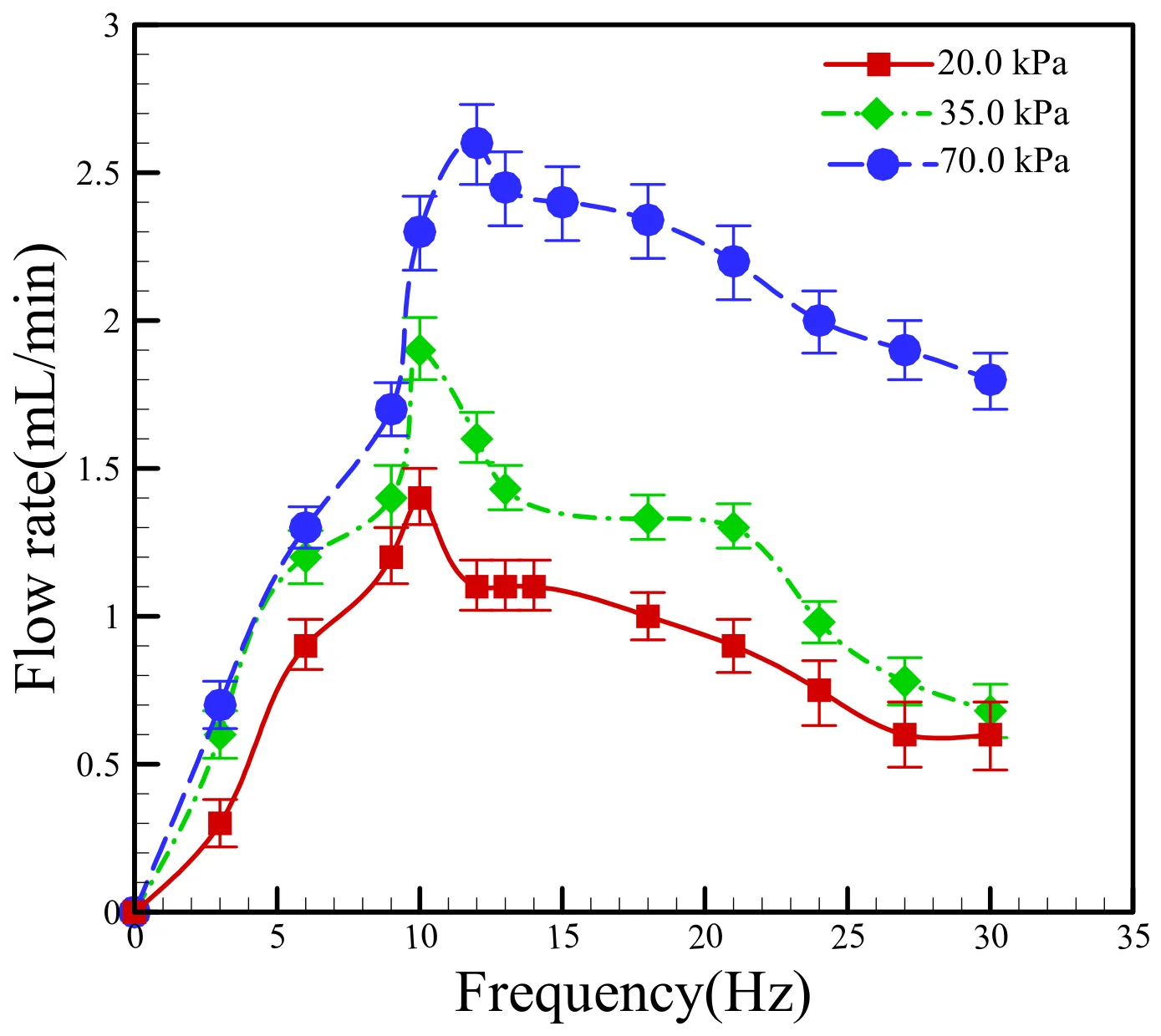
Experimental volumetric flow rate versus operating frequency under various pneumatic pressures. Error bars indicate standard deviations based on measurements from three independent samples across six testing runs.

Figure 10 illustrates the experimentally measured volumetric flow rate of the proposed micropump across various driving frequencies at 20.0, 35.0, and 70.0 kPa. The experimental data exhibit a characteristic bell-shaped frequency response. In the low-frequency regime (<10.0 Hz), the pneumatic charging and discharging times are clearly sufficient for the membrane to achieve its maximum stroke volume (ΔV); thus, the overall flow rate increases linearly with the driving frequency (Q = f∙ΔV). However, as the frequency surpasses an optimum threshold (~10.0–12.0 Hz), the flow rate sharply attenuates. This high-frequency degradation occurs because the pneumatic period becomes shorter than the system’s inherent time constant, which prevents the membrane from reaching its full stroke before the cycle reverses.

Furthermore, a subtle shift in the optimum frequency is observed (i.e., from ~10.0 Hz at 20.0 and 40.0 kPa to 12.0 Hz at 70.0 kPa). This positive correlation can be explained by the nonlinear structural mechanics of the membrane. Under a high pressure (70.0 kPa), the large deflection enters a stretching-dominated regime, resulting in structural strain stiffening. This increased stiffness effectively reduces the fluidic compliance (C). According to the lumped-parameter time-constant model (τ = RC, where R is fluidic resistance), a reduced compliance yields a smaller time constant, resulting in a higher critical resonance frequency. Additionally, at 70.0 kPa, the membrane makes physical contact with the 1.5-mm chamber bottom. Overcoming the subsequent squeeze-film damping and suction effects during the upward recovery stroke notably contributes to the flow-rate attenuation observed in the high-frequency bounds.

**Figure 11:**
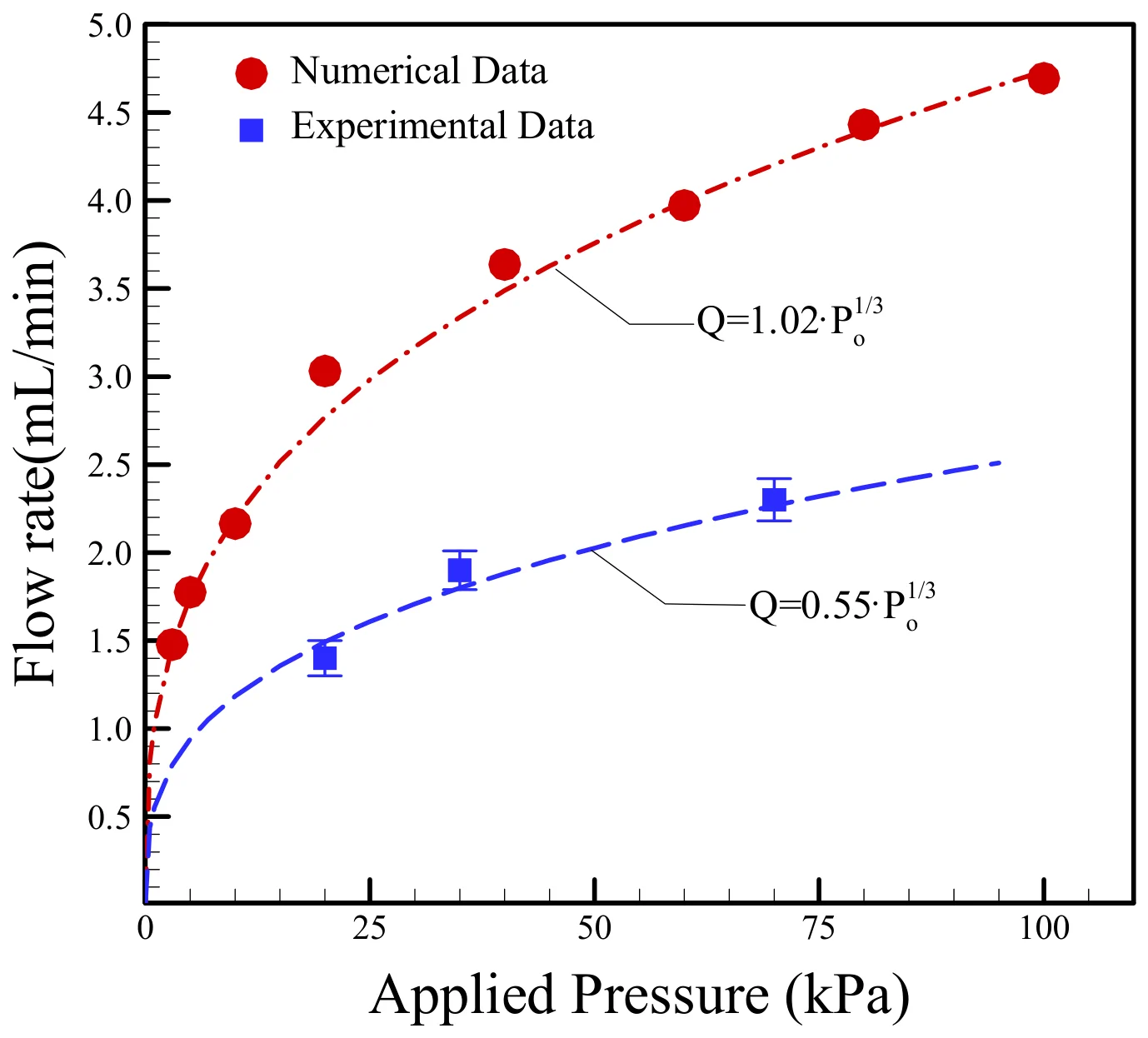
Comprehensive comparison of the experimental and simulated volumetric flow rates of the micropump at a driving frequency of 10.0 Hz.

Under these idealized conditions (synchronized wall-switching), the ideal upper-limit volumetric flow rates for both suction and discharge strokes were determined through a rigorous stepwise calculation. First, the instantaneous spatial-averaged velocity at each time step was obtained by integrating the transient velocity field over the entire port cross-section (1.5 × 1.5 mm^2^) and dividing by the cross-sectional area. Subsequently, the overall time-averaged velocity was derived by integrating these instantaneous velocities over the active phase duration and dividing by the half-cycle period. Finally, the time-averaged volumetric flow rate was calculated by multiplying this overall time-averaged velocity by the cross-sectional area. This precise calculation procedure fully accounts for the spatial velocity distribution within the square channels and ensures strict mass conservation. Figure 11 presents a comprehensive comparison of the volumetric flow rates obtained from the 3D FSI numerical simulation and experimental measurements at 10.0 Hz. Within this established framework, the numerical simulation serves as the idealized flow rate (the theoretical upper limit), whereas the experimental validation represents a much more realistic physical scenario. Based on the curve-fitting analyses of both the numerical and experimental data, the relationship between the volumetric flow rate and the applied pressure can be expressed as a general form:

(12) $$Q=C\cdot P_{o}^{1/3}$$

where Q is the volumetric flow rate (mL/min), and P_o_ is the applied pressure (kPa). The empirical coefficient C is determined to be 1.02 for the numerical simulation data and 0.55 for the experimental measurements, respectively, with the experimental data consistently yielding lower flow rates than the idealized simulation.

To quantify the practical performance of the system relative to the ideal numerical baseline, the pump efficiency (η) is defined as the ratio of the experimental flow rate to the simulated flow rate:

(13) $$\eta =\frac{Q_{\exp}}{Q_{sim}}\times 100\%$$

where *Q*_exp_ and *Q_sim_* denote the volumetric flow rates obtained from experimental measurements and numerical simulations, respectively. Based on Equation (13), the proposed micropump demonstrates a pumping efficiency of 50.57% at 20.0 kPa, peaking at 56.95% at 35.0 kPa, and slightly decreasing to 54.71% at 70.0 kPa.

The discrepancy between the ideal upper limit (e.g., simulation) and the experimental flow rates stems primarily from real-world system inefficiencies. Specifically, two practical factors contribute to this efficiency loss: (1) PCV dynamic leakage: Unlike the instantaneous wall-switching assumed in the model, physical check valves experience a microsecond closing delay upon pressure reversal, resulting in internal backflow and dynamic leakage. (2) Parasitic system compliance: The microscopic expansion of the soft PDMS layers and flexible connecting tubing under high pressures absorbs a portion of the pneumatic energy, dampening the effective stroke volume.

## 5. Conclusions

This study comprehensively evaluates a pneumatic PDMS micropump via rigorous 3D FSI numerical simulations of an incompressible Newtonian fluid, combined with experimental analyses. By bridging numerical upper limits with practical fluidic losses, this validated framework serves as a robust predictive tool for optimizing flow asymmetry and net pumping efficiency in advanced microfluidic systems. The key findings are summarized as follows:

(1) 3D FSI modeling and asymmetric dynamics: A robust FSI simulation model was successfully established to elucidate the complex coupling mechanism between large membrane deformations and the internal flow field. The simulations revealed significant asymmetric dynamics. Notably, when the applied pressure exceeds 20.0 kPa, the discharge flow rate becomes substantially greater than the suction flow rate.

(2) Structural deformation validation: The 3D FSI numerical results were compared with the classic Timoshenko formula for predicting the maximum membrane deformation, demonstrating excellent agreement across the entire pressure range (3.0–100.0 kPa). Notably, the relative deviation between the simulated results and theoretical predictions remained strictly below 3% under all the tested conditions.

(3) Experimental peak performance: The proposed micropump demonstrated a maximum volumetric flow rate of 2.6 mL/min at 70.0 kPa and 12.0 Hz. When evaluated at a constant driving frequency of 10.0 Hz, the pump achieved flow rates of 1.4, 1.9, and 2.3 mL/min at 20.0, 35.0, and 70.0 kPa, respectively.

(4) Numerical and experimental performance evaluation: Curve fitting of the numerical and experimental data reveals that both flow rates follow the relationship $Q=C\cdot P_{o}^{1/3}$, where P_o_ is the applied pressure (kPa) and Q is the flow rate (mL/min). The empirical coefficient C was 1.02 for the idealized numerical baseline and 0.55 for the experimental validation, respectively. Furthermore, the pumping efficiency of the microfluidic system was evaluated as 50.57%, 56.95%, and 54.71% at 20.0, 35.0, and 70.0 kPa, peaking at 35.0 kPa.

## Figures and Tables

**Table 1:** Geometrical specifications of each part for the proposed micropump.

Index	Section	Geometrical Dimension (mm)			
		Diameter	Length	Width	Thickness
1	air chamber	10.0	─	─	1.0
2	actuated membrane	─	30.0	20.0	0.3
3	fluidic pump chamber	10.0	─	─	1.5
4	fluidic Channel 1	─	5.0	3.0	1.5
5	fluidic Channel 2	─	5.0	1.5	1.5
6	passive check valve		0.5	2.8	1.4

## Data Availability

The data presented in this study are available within the article. Additional data that support the findings of this study are available from the corresponding author upon reasonable request.

## Supplementary Materials

The Supplementary Document S1 and Video S2 are openly available in Zenodo at https://zenodo.org/records/22104620, accessed on 25 August 2026.
